# Perinatal Mental Health Care for Women With Severe Mental Illness During the COVID-19 Pandemic in India—Challenges and Potential Solutions Based on Two Case Reports

**DOI:** 10.3389/fgwh.2021.648429

**Published:** 2021-07-14

**Authors:** Sachin Nagendrappa, Pratibha Vinod, Naveen Manohar Pai, Sundarnag Ganjekar, Geetha Desai, M. Thomas Kishore, Harish Thippeswamy, Kimneihat Vaiphei, Prabha S. Chandra

**Affiliations:** ^1^Department of Psychiatry, National Institute of Mental Health and Neurosciences, Bangalore, India; ^2^Department of Clinical Psychology, National Institute of Mental Health and Neurosciences, Bangalore, India; ^3^Department of Psychiatric Social Work, National Institute of Mental Health and Neurosciences, Bangalore, India

**Keywords:** severe mental illness, COVID-19, challenges, virtual care, perinatal mental health

## Abstract

The ongoing COVID-19 pandemic in India has created several challenges in the care of women with perinatal mental illness. Access to healthcare has been disrupted by lockdowns, travel restrictions, and the unavailability of outpatient services. This report aims to discuss the challenges faced by women with severe mental illnesses during the perinatal period with the help of two case reports. Accordingly, we have highlighted the role of COVID-19 infection as a traumatic event during childbirth and its role in triggering a psychotic episode in women with vulnerabilities; difficulties faced by women with postpartum psychosis in accessing perinatal psychiatry services; and the challenges of admission into an inpatient Mother-Baby Unit (MBU). Further, we have discussed potential solutions from the perspectives of Lower and Middle-income (LAMI) countries that need to be extended beyond the pandemic. They include offering video consultations, reviewing hospital policies, and evolving strategies to mitigate traumatic experiences for pregnant and postpartum women with severe mental illnesses in both obstetric and psychiatric care.

## Introduction

Pregnancy and postpartum in women are vulnerable periods for developing severe mental illness (SMI) or exacerbating pre-existing psychiatric illnesses (1, 2). Globally, the COVID-19 pandemic has significantly affected the clinical care of patients with mental illnesses particularly those with severe mental illness. High-income countries have made necessary modifications which include reorganization of services for persons with SMI, early discharges, reduced ratio of the number of mental health care staff per inpatient in psychiatric hospitals, and the use of telepsychiatry services to cater to the population's mental health needs (3–5).

In India, the first case of COVID-19 was detected on 3rd February 2020. By mid-September 2020, India reached the peak of the first wave with 93,198 cases. At the height of the 1st wave, India had 0.85 deaths per million people. After that, there was a declining trend noted until 17th February 2021. The second wave hit the country in March 2021. At the time of this report, May 2021, India has the 2nd most active COVID-19 cases globally, with 2.31 deaths per million people due to COVID-19. During the last weeks of April 2021, India reported more than 3.5 Lakh cases in a single day. As of now, there is no official data on pregnant and postpartum women contracting COVID-19. As per the latest data on 3rd May 2021, India has 2,02,92,331 confirmed cases, among which 34,51,826 were active cases and 2,22,478 deceased due to COVID-19 (6, 7).

Like many other countries, India faced challenges in caring for people with mental illnesses due to specific changes imperative to the COVID-19 pandemic. These included the nationwide lockdown, which was imposed from 25th March 2020 to 30th May 2020. The lockdown necessitated travel restrictions and the closure of psychiatric outpatient services. In addition, several general hospitals were converted to COVID-19 designated hospitals to meet the increasing demand. As published reports indicate, psychiatric services were reorganized, whereby patients were primarily seen in emergency settings rather than in outpatient settings while being highly selective with inpatient admissions (8–10). However, there is no data on how perinatal psychiatric services were affected during the COVID-19 in LAMI countries.

The psychological impact of the COVID-19 pandemic on women during pregnancy and the postpartum period has been relatively high. Globally, evidence shows an increase in anxiety and depression and thoughts of self-harm among women in the perinatal period (11, 12). Fears due to pandemic-related measures like quarantine and isolation; and risk of exposure to infection and fetal transmission are a few factors related to the high psychological morbidity (11, 13, 14). Changes in the schedule of appointments, restrictions related to birth companions and a decrease in the number of postnatal visits have been other contributing factors (12). However, most of the data on COVID-19 related perinatal mental health problems focuses on common mental disorders such as anxiety and depression. There is limited literature on the challenges faced by women with severe mental illness in pregnancy or postpartum during the pandemic. Additionally, studies have shown a rise in intimate partner violence during the lockdown emphasizing the need for adequate and appropriate services (15–17).

Disruptions in healthcare due to disasters are common and the immense psychological distress caused in such circumstances is often overlooked. For example, a longitudinal study (18) from Japan highlights how the emotional upheaval caused by the Tsunami affected mothers' mental health and the struggle they went through to get appropriate help. Psychological distress continued to be prevalent up to 4 years after the disaster and common issues related to distress were economic problems, dissatisfaction in the marital relationship, and no support with childcare. A study from Nepal (19) indicates that there was an increased risk of common mental disorders among pregnant and postpartum women during the aftermath of an earthquake. The study also noted an increase in adverse birth outcomes. The study further suggests prioritizing the mental health of pregnant women in post-disaster management because of the burden experienced by the women and the associated risk to the growth and development of their babies. However, there is no data to draw parallels on the implications of COVID-19 for women with postpartum severe mental illness. The current report discusses through case examples, the challenges that women faced and how the perinatal psychiatric services in a LAMIC setting changed to accommodate the emerging needs of women with SMI during postpartum.

### Case Study: Setting

The two women being described sought help from the Perinatal Psychiatric Services at the National Institute of Mental Health and Neurosciences (NIMHANS), Bengaluru, India. The Perinatal Psychiatric Services include an exclusive weekly outpatient clinic for women with perinatal mental health problems and an inpatient Mother-Baby Unit (MBU). The clinic receives an average of 35–40 women every week, and the MBU has an average of 60–70 admissions a year (20).

The perinatal outpatient clinic and the MBU had to be closed as soon as the pandemic started. The MBU unit remained closed for 2 months, following which it was opened with restricted number of admissions. Outpatient services remained shut throughout. In the perinatal period, most women with SMI were attended to by emergency psychiatric services and a brief admission of 48 hours in a short stay ward. Women could reach the perinatal psychiatry services using a pre-existing perinatal helpline as well. Depending on the clinical severity, they were either admitted to the MBU or treated in the community setting, a preferred option. However, every attempt was made to liaise with the obstetric care services throughout pregnancy and postpartum. The two case reports described in the next section were chosen to describe challenges at two different points of care, i.e., the emergency psychiatry service and in the MBU. They represent similar experiences faced by other pregnant and postpartum women during this period.

### Case 1

A woman in her thirties who tested positive for COVID-19 infection in July 2020, 4 days before her due date for delivery was referred for psychiatry care. She had no physical symptoms of COVID-19, such as fever, breathlessness, cough, or sore throat. She was admitted to a COVID-19 designated hospital for delivery instead of the hospital where she had earlier registered for delivery and received antenatal care. The baby was delivered in the COVID-19 designated hospital through an elective Cesarean section because of cephalo-pelvic disproportion (CPD) diagnosed earlier. Following the delivery, the infant was kept away from the mother for 5 days in the hospital nursery, and she was not allowed to breastfeed as per the hospital's policy at that time.

The infant was shown to her over video calls by a relative and the nursing staff. While speaking over the phone to her family, she described feeling isolated in the hospital, worried about not being able to breastfeed the baby, and feeling neglected by the hospital staff. She was discharged from the hospital on the 5th day postpartum and was advised home quarantine for the next 2 weeks as per government guidelines. All clinical decisions (i.e., the infant being kept away from the mother for 5 days, not being permitted to breastfeed or give expressed milk) were in line only with the hospital's protocol where she delivered. Available guidelines by WHO and others during that time however, did not restrict breastfeeding among mothers with COVID-19 infection or rooming-in (21–24).

On the tenth day postpartum, she started expressing guilt for having contracted COVID-19 and inconveniencing family members. She was afraid and anxious that she would transmit the COVID-19 infection to the infant and family members and had left home on one occasion saying that the infant was better off without her. However, the family grew concerned when they observed that she poured water over herself for no reason, started neglecting the infant, stopped caring for herself, and expressed ideas of self-harm. The family knew about the perinatal psychiatry helpline as she had been treated for a previous episode of postpartum psychosis and contacted the service.

During the previous postpartum period, she was diagnosed to have Psychosis NOS when she reported suspiciousness, hearing voices, and irritability. As a result, she was on Risperidone 4 mg for 3 years, after which she stopped medication on her own. However, she was well during her pregnancy.

Through video calls, patient was assessed by our team, and an ICD-10 diagnosis of Acute Polymorphic Psychotic disorder- without symptoms of schizophrenia (ICD Code-F23.0) with postpartum onset was made. In addition, a differential diagnosis of Organic Psychotic Disorder was considered keeping in mind possible neuropsychiatric manifestation of COVID-19. However, there was no history of delirium or symptoms and signs of an organic disorder.

It appeared that the combination of a COVID-19 diagnosis, the subsequent isolation, and separation from the infant was a traumatic experience and a possible triggering factor for postpartum psychiatric illness. She was advised inpatient admission. However, the family requested home-based care.

#### Challenges in the Inpatient Care and Mother-Infant Care

Mental status examinations had to be done via video calls. It was a challenge to rule out organicity through video calls, and a physical examination was not possible. Her progress was reviewed daily over video consultation by the senior resident in the team for a week and weekly once thereafter. The assessments focused on symptoms of psychosis, anxiety, and PTSD as well as any risk of harm to self or infant. Given the good initial response to Tab. Risperidone, she was prescribed the same up to 6 mg. She showed gradual improvement over 2 weeks and was encouraged to breastfeed her infant using all precautions. However, she continued to be anxious about spreading COVID-19 infection to her infant.

Maternal-infant bonding assessment and infant's developmental assessment were also done over video calls. Overall, based on observation, informal review, and parental reports, the infant had age-appropriate development, and mother-infant bonding gradually improved.

While all attempts were made to use telepsychiatry for infant assessment and mother-infant bonding which are essential components of care when the mother has postpartum psychosis, these were difficult to conduct. Postpartum psychiatric illnesses affect mother-infant bonding and warrant early interventions to prevent long-term effects (20, 25, 26). Assessment of infants for attention, object tracking, play, and other parameters of cognitive development is challenging to conduct virtually, and clinicians need to rely on the information given by the family. Also, one cannot expect the infant always to face the phone screen during a virtual assessment. In our dyadic assessment, appointments were canceled twice due to the infant's immunization schedule, and the infant was asleep. Technical difficulties in the evaluation included not getting the mother and the baby in one frame and frequent interruptions due to poor internet connectivity.

#### Overcoming the Barriers

Many of the challenges were handled by teleconsultations (video calls over the phone). Prescriptions were sent online, and when in doubt, the family contacted us through the perinatal phone helpline. A virtual physical examination using questions to rule out organicity was performed. Infant's development and mother infant bonding were assessed over video calls, and weekly follow-ups ensured contact with the dyad and the family.

### Case 2

Our second case is a 25 year old woman who sought help for relapse of her mental illness in the postpartum following an unplanned pregnancy. She had been receiving treatment for bipolar affective disorder, and the current episode, which started in the second trimester, worsened on the 20th day of the postpartum. This episode was precipitated by treatment non-adherence for 2 weeks as the patient was unable to follow up in the outpatient due to travel restrictions and had stopped medications as they were not available during the lockdown. In addition to Bipolar Disorder, she also had several personality traits such as impulsivity and sensitivity to criticism which added to the clinical problems. She had been prescribed Tab. Lithium 900 mg and Tab Risperidone 2 mg, but she was irregular with her medications after fights with family members. Two weeks after stopping the medication during the lockdown, she started showing several symptoms such as irritability, increased energy levels, overfamiliarity, disinhibition, overspending, overgrooming, elevated self-esteem, decreased need for sleep, and multiple delusions of infidelity, reference, and persecution. She had also made suicidal attempts of high intentionality and lethality.

She delivered a male baby in a charitable health care center, where no psychiatrist was available. Her manic symptoms were not identified or treated, and gradually she became unmanageable at home. In the emergency psychiatry service, the initial assessment revealed a high risk of harm to self and others. Patient and her caregiver were required to take the COVID-19 test in the emergency ward before inpatient care as per hospital protocol. The infant had to be under the supervision of his grandmother because she was unable to care for him and the family was concerned about COVID 19 infection. She was admitted to the MBU (which had reopened in July 2020) but left against medical advice as she found it difficult to follow COVID 19 appropriate behavior. She was brought back by family members on the second day as she remained challenging to manage at home. The treating team decided on providing electroconvulsive therapy (ECT) because of high suicidal risk. She was given six bifrontal ECTs on alternate days, with threshold ECT charge being 120 mC, Pulse amplitude 800 mA, pulse width 1.5 ms, pulse pair frequency 62.5 Hz, average motor seizure duration 30 s. She showed improvement following the ECTs. The hospital had developed a specific protocol for ECTs for the COVID 19 pandemic which included organization and training of ECT services team such as mandatory COVID-19 testing before initiating ECTs, use of personal protective equipments, modifications in ECT administration and recovery areas, use of heat and moisture exchanger (HME) filters, appropriate cleaning and disinfection (27).

In the MBU, where she was admitted with her mother in law as a caregiver (who also had to be tested for COVID 19 before admission), she was disinterested in infant care and had poor mother-infant bonding. The team planned mother-infant bonding interventions, but within a few days, she had to be categorized at high risk of exposure to COVID-19 after another patient in the adjacent ward tested positive for the virus. Due to her symptoms, she would insist on interacting with other patients without a mask and without keeping social distance. The family was worried about the possibility of COVID-19 exposure due to her behavior and requested early discharge despite only partial clinical improvement and poor bonding with the infant.

#### Challenges in Inpatient Care and Mother-Infant Care

The woman had to undergo repeated testing for COVID-19 infection, which caused a lot of stress and inconvenience for both the patient and the family. Her admission was delayed due to the protocol of mandatory testing for COVID-19 for inpatients as well as any caregivers who stayed with them. After being discharged from the hospital, she found it difficult to come for follow-ups due to travel restrictions.

#### Overcoming the Barriers

Despite the interruption in her routine inpatient care, the family continued to stay in touch with the service over the perinatal psychiatry helpline. However, mother-infant bonding and infant care continued to be poor, and the infant had to be under the supervision of his grandmother. The family was advised to bring the patient and infant for mother-infant bonding and developmental assessment on an outpatient basis in the perinatal clinic. However, she did not adhere to the advice due to fear of COVID-19. Telephonic follows up were done with the grandmother, who reported that the infant was doing well and was on formula feeds as the mother did not want to breastfeed.

## Discussion

Any natural disaster can have cross-sectional and longitudinal implications for the mental health of pregnant and postpartum women. However, research focusing on perinatal women with severe mental illness is scarce. The two cases described above exemplify the challenges faced by women who need perinatal mental health services during the pandemic and how the system may adapt itself to suit these needs. With the COVID-19 pandemic, numerous unforeseen barriers have emerged which have negatively impacted the mental health of pregnant and postpartum women. Having a severe mental illness itself causes many challenges, and when a crisis like COVID-19 happens, the challenges become more complex. A survey conducted in the UK to understand the perception of mental health staff who closely worked with women with mental health problems in the perinatal period has highlighted some critical concerns (28). The survey revealed that lack of access to usual support networks, feeling lonely, lack of work and activities, worries about contracting COVID-19 infection, poor access to mental health services, increased risk from abusive domestic relationships, and the risks of relapse were significant concerns. We have observed and faced similar circumstances in our MBU in India, as highlighted by the two case reports.

Perinatal psychiatric services include outpatient services and MBU, which are dedicated to women with severe mental illnesses. Studies have shown that mother-infant dyads admitted to MBUs have good clinical outcomes and low readmission rates (20). MBUs provide holistic services ranging from pre-conception counseling to handling mental health problems during pregnancy and postpartum and providing dyadic interventions. While most high-income countries have these facilities, such specialized services are scarce in most LAMI countries, where the mental healthcare systems are often fragmented. The current pandemic has worsened the situation, and it has been reported that many women have had no access to medical termination of pregnancy or contraceptive services during lockdowns (29) which might have led to unplanned pregnancies in women with severe mental illness. Besides, there has been an inadequate supply of medications for those with pre-existing psychiatric problems and ineffective communication between mental health providers and women with mental illnesses of the child-bearing age. Existing literature also suggests a rise in intimate partner violence in women during the COVID-19 pandemic, which adds to the challenges of providing optimum mental health care and increases the risk of postpartum mental health problems (30).

The COVID-19 task force of the Research Innovation and Sustainable Pan-European Network in Peripartum Depression Disorder (RISEUP-PPD) report discusses good practices in perinatal mental health care during the COVID-19 pandemic (12). The report includes providing information about psychological issues and ensuring adequate social support involving partners and immediate caregivers in the care of mothers during pregnancy and childbirth. The report also emphasizes the need for identifying and facilitating sources for help, case detection, and emotional support for women facing intimate partner violence in the postpartum period. While the International Marcé Society for perinatal psychiatry has developed a series of COVID-19 related resources, most of these are about women with anxiety, depression, or trauma-related symptoms (31). Not much has been written about pregnant or postpartum women with severe mental illnesses and their care during the COVD-19 pandemic, especially in LAMI countries.

## Potential Solutions Which Help During the Pandemic Must Continue Beyond the Pandemic

There is a need for “women friendly” hospital policies focusing on COVID-19 and for the post-pandemic period to identify, screen and treat common and severe mental illness during pregnancy and postpartum. Appropriate changes are needed at the policy level for women at risk of mental health problems, which include maternity hospitals ensuring the presence of caregivers during delivery and postpartum; adequate counseling to the mothers if they have COVID-19 infection; and sensitive guidance regarding rooming-in and breastfeeding keeping the interest of the mother-infant dyad. There is also a great need to integrate mental health services in existing COVID-19 related obstetric health care services (32, 33). Guidelines and policies in obstetric units should emphasize liaison with mental health professionals to address women's mental health concerns in the perinatal period, especially for those women who have pre existing mental health problems and are more vulnerable. Priority should be given to provide prompt COVID-19 test results for postpartum mothers in psychiatric services so that they can be provided inpatient care early. All the above measures may help prevent excessive trauma and stress, especially in vulnerable women. Obstetricians, midwives and pediatricians need to be trained in early identification and there is a need to strengthen referral services.

### Trauma-Informed Care

In addition to the usual perinatal mental health problems that one sees, there has been an increase in trauma-related symptoms during the pandemic. Hence, there is a need to evolve trauma-informed services in MBUs (34, 35). The goal is to address fear, anxiety, stress, grief, and other signs of psychological distress and provide psychological support in COVID-19 care maternity wards.

### Virtual Interventions and Perinatal Phone Helplines

In LAMI countries like India, there is a significant treatment gap for persons with mental illness, which has worsened during the pandemic. The COVID-19 pandemic has taught us that this gap could be bridged to some extent by using technology (36). However, technology comes with its own set of unique challenges such as internet connectivity issues, privacy problems when women live in joint families and crowded housing, and problems related to virtual assessment of mother-infant dyadic relationships. There is a need to evolve telepsychiatry protocols for helping women with infants living in remote locations, in low-income settings, and those facing IPV (37). Virtual interventions have shown promise in delivering care adequately to perinatal women with depression and anxiety. However, their role in women with postpartum psychosis is still not very clear (38, 39).

Telephonic helpline services provide a more accessible alternative for women to access health care even in LAMI countries. Through helplines, women can access information, identify and manage symptoms, and seek help when necessary, including post-discharge care (40). For example, a study from our MBU in India prior to the pandemic found that a Perinatal Mental Health helpline is useful in addressing a wide range of concerns including medication schedules and side effects, symptom exacerbation, suicide risk, pre conception planning, breastfeeding problems, and seeking appointments (41). Started much before the COVID-19 pandemic, in 2016, the already existing perinatal phone helpline was an extremely useful resource for women with SMI during the pandemic, as exemplified in the cases discussed above.

## Conclusions and Future Directions

Mental health implications of the COVID-19 pandemic for pregnant and postpartum women are comparable to other disasters. However, the COVID-19 pandemic created a different set of challenges because of the risk of infection, the prolonged nature of the pandemic and inability to access resources due to lockdowns and travel restrictions. It led to a decline in women accessing care for perinatal mental health problems and this had a negative impact especially among those with SMI. The pandemic has also exposed the inadequacies of liaison between obstetric and mental health care systems in LAMI countries and revealed pre-existing deficiencies in the care of pregnant and postpartum women with mental illness. Immediate attention is needed to support postnatal mothers and provisions made for continued mother-infant interactions. There is also a need for revisiting hospital policies that restrict the physical presence of caregivers during childbirth in obstetric units. Attempts must be made to enhance care, security, and safety which may alleviate traumatic experiences and psychological distress during and following childbirth. In psychiatric settings, special consideration must be given to mothers with infants for rapid testing and reporting for COVID infection to prevent delays in admission and intervention. There is also a need for further research in the most appropriate methods for virtual assessments of mental status of women with postpartum psychosis, mother-infant interaction and infant development and addressing the needs of perinatal women facing domestic violence.

## Ethics Statement

Written informed consent was obtained from the individuals for the publication of any potentially identifiable images or data included in this article.

## Author Contributions

All authors listed have made a substantial, direct and intellectual contribution to the work, and approved it for publication.

## Conflict of Interest

The authors declare that the research was conducted in the absence of any commercial or financial relationships that could be construed as a potential conflict of interest.
